# Communication skills learning through role models in Nepal; what are medical students really learning? A qualitative study

**DOI:** 10.1186/s12909-021-03049-0

**Published:** 2021-12-20

**Authors:** Amanda Helen Douglas, Samita Pant Acharya, Lynne A. Allery

**Affiliations:** 1 Department of GP, Patan Academy of Health Sciences (PAHS), Lalitpur, P.O.Box 26500, Kathmandu, Nepal; 2 Reader in Medical Education, Centre for Medical Education, Cardiff University, Heath Park, Cardiff, CF14 4YS UK

**Keywords:** Communication skills, Medical education, Nepal, Role-modelling, Students

## Abstract

**Background:**

Communication skills (CS) are important and teachable, however, many Asian medical schools’ curricula do not incorporate them. Patan Academy of Health Sciences in Nepal identifies CS within its’ aims and curriculum. CS are taught from commencement of medical school and re-emphasised throughout preclinical learning (first 2 years). There is no explicit CS teaching in clinical years but placements allow students to learn through observation. These ‘role-modelling’ interactions form part of CS learning and development.

**Methods:**

This study is a qualitative evaluation of CS learning in PAHS, through participants’ experiences. Through purposive sampling, twenty medical students from 2nd, 4th and Intern years were selected for inclusion. Data were collected via audio recorded, semi-structured interviews, employing a piloted schedule. Transcripts were manually coded and analysed thematically. Codes were organised into themes and subthemes. This paper discusses themes related to role-modelling.

**Results:**

The majority of participants described role-modelling in CS learning, recounting both positive and negative incidents, reflected in the themes of; Positive and Negative experiences. Subthemes of Personal Qualities and Inspiring, emerged from positive experiences, describing students’ desire to imitate or aspire to be like their role models. Learners reported predominantly negative experiences and interns exclusively so. From these emerged subthemes of; Good doctors but.., Contradictory messages, How not to behave, Unprofessional behaviour and Affect-Emotional Distress. Learners received conflicting messages from observing behaviour contradictory to explicit CS teaching. Many identified learning “how not to behave” from such incidents, however, several described feeling distressed.

**Discussion:**

Role-modelling is a powerful and important CS learning tool, seen as positively reinforcing or negatively contradicting explicit CS teaching. Negative modelling created internal conflict, confusion and distress amongst learners, despite its’ potential for positive learning. The worldwide problem of negative role-modelling is also prevalent in Nepal.

Medical educators need to ensure the explicit curriculum aligns with implicit learning. Clinical tutors must be alerted to their powerful role-model position and supported in developing intentional modelling skills. Learners’ reflections upon their experiences should be facilitated, enabling them to critically evaluate observations and hence consciously adopt or reject role-modelled behaviour and attitudes.

## Background

Communication skills are core competencies for both doctors and medical students [1, 2]. Good doctor-patient communication is seen to positively impact almost all aspects of patient care [3–7]. Communication may be regarded as the “main ingredient” of medical care [5]. In contrast, poor communication negatively impacts patient understanding [8, 9]. The communication skills required for doctors are teachable [10] and included within the majority of western medical schools’ curricula [11, 12]. However, despite their inclusion within medical teaching, good CS are often lacking in patient care [13, 14]. Evidently some doctors fail to acquire the skills or attitudes needed to communicate well [15].

CS teaching is relatively new in South Asia. Over 20 years ago CS were highlighted as core skills for Indian medical graduates [16], however, very few medical schools implemented its’ teaching [17, 18] until 2015, when MCI introduced an attitude and communication module [19] across the country [18]. In Nepal, as in India, CS teaching is in its’ infancy. A 2006 a report [20] stated that the majority of Nepalese medical schools’ did not include CS, doctor-patient relationship or ethics within their curricula. These have since been identified within the Nepal Medical Councils’ core competencies [21] and several medical schools have introduced CS teaching [20, 22, 23].

Patan Academy of Health Sciences (PAHS), Nepal is an independent, non -profit institution, founded in 2010 primarily to train health workers for rural Nepal. It seeks to train; “*competent, caring and socially responsible physicians…….who*; *Believe in compassion, love, respect, fairness and excellence*. *Communicate well with patients, family and colleagues*”. In line with this, PAHS’ has incorporated formal CS teaching from early in the curriculum. The PAHS’ medical course is divided into the preclinical (basic science) course, of 2.5 years and clinical course of 2 years. This is followed by a 1-year student internship. The Pre-clinical curriculum includes a mixture of didactic teaching and problem-based learning, together with a twice weekly Introduction to Clinical Medicine Course (ICM), which facilitates students’ early exposure to the clinical environment. Explicit, structured CS teaching is conducted within the ICM course, throughout the pre-clinical years. The CS curriculum includes; listening, information giving, angry patient management, breaking bad news and general communication skills. All General Practice faculty, who have undergone CS training, coordinate and conduct both undergraduate and postgraduate CS teaching. None of the GP faculty have a special interest in medical education and there is little formal educational training for staff development although several have undertaken one-day courses on ‘how to teach’. CS sessions incorporate both lectures and roleplay. No formal, explicit CS teaching occurs in the clinical years, however, students are expected to practice and build upon their skills during clinical placements. CS comprise components of both preclinical and clinical years assessments through; clinical evaluation exercises (Mini-CEX), OSCE (Objective Structured clinical examinations), direct observation of procedural skills (DOPS) and end of placement assessment. Medical education is primarily conducted in English; however, community and patient interactions are usually undertaken in Nepalese. Clinical placements enable students to observe and interact with senior doctors in practice, undertaking medical duties and interact with patients. This may be termed ‘role modelling’, the means through which teachers “demonstrate clinical skills.. and manifest positive professional characteristics” [24].

### Role Modelling

Role modelling is a significant and usually implicit component of most medical school training [25]. A role model is any individual who teaches or inspires by example whilst engaged in something else [26]. Role models function anywhere that a clinical teacher is observed by a student [27]. Either in a planned intentional process [28] or, as usually occurs, in an informal and unplanned manner, with students learning directly through observation [29]. Role modelling is powerful. Students learn through unceasing observation, noticing particularly interactions with others [30], they adopt the beliefs and behaviors, values and ideals of the medical profession they have observed [31–34]. Role models have a strong influence [35] and are a significant factor in the development of patient-centred values [36] and CS.

Students hold a position of “legitimate peripheral participation” within the medical “community of practice” [37]. Learners interactions and observations from this position shape, not only their behaviour and ideas of what it means to be a professional, but also their professional identity- how they consider themselves as doctors [37, 38]. Through socialisation, learners adopt the technical skills, practices and values of the community and culture around them [39]. This is a vital part of the development of the student into a medical professional [40, 41]. Role modelling is a form of socialisation [38] and has a profound impact on students’ professional identity formation [42–44].

PAHS is a relatively new medical school whose CS curriculum is unique in Nepal. This study comprises part of a qualitative CS curriculum evaluation. Participants’ experiences and perceptions of this CS curriculum were explored in an earlier study [45]. This paper seeks to explore students’ experiences of CS learning through role modelling in this context.

## Methods

This study employs qualitative methodology, an interpretivist paradigm and subjectivist, social construction epistemology. The study intends to understand the student participants’ subjective reality and their experiences [46]. Semi-structured interviews enabled in depth exploration of participants responses and the freedom to consider different issues [47] as the interview was co-constructed by researcher and participant together [48].

### Participants

The study population comprised of second year (basic science course), fourth year (clinical course) and Intern year medical students from PAHS. In order to ensure diverse perspectives were explored students were taken from several cohort years, with different degrees of clinical exposure and proximity to preclinical formal CS teaching. Purposive sampling was employed. An email invitation to participate was sent to all 60 students within each study year group, via student representatives. Interviews were arranged and conducted, commencing with the first responders, until data saturation indicated a sufficient number had been completed [49].

Twenty undergraduate and Intern medical students were interviewed; seven from the 2nd and 4th years and six student Interns. Interviews were conducted between February and April 2018.

### Ethical consideration

Local ethical approval was obtained from the Patan Hospital ethics review board, prior to commencement. All participation in the study was voluntary and informed written consent was taken. Participants were aware that they were free to decline to participate or withdraw prior to thematic analysis. Participants understood their identity would remain confidential and views and opinions would be anonymised. The interviewer was a volunteer teacher at PAHS and hence known to many participants, however, her teaching of the participants’ year groups was limited and she was not involved in their communications assessments. Participant interviews were allocated a code number and pseudonym, to maintain cultural appropriacy, used in all reporting. All identifying features were removed during transcription. Participants understood that there were no correct answers and that all their opinions and comments were welcome. Participants were aware that the interviews constituted part of a research project, may be published and used to improve medical education.

### Reflexivity

All interviews were undertaken by the first author, a female, British doctor, resident in Nepal and member of GP faculty, and supervised as part of a postgraduate Cardiff University qualification. Thorough preparation was done before data collection as the interviewer was new to qualitative research. Interview skills and technique were practiced through pilot interviews. Reflexivity was incorporated within the study as the researchers’ perspective is integral to findings [50]. A field note journal was employed for the researcher to reflect upon her thoughts, feelings and impressions and consider their impact on the data generated [51].

### Data collection

Twenty face-to-face, semi-structured interviews were conducted. Each lasted from 20 to 45 min and were undertaken at a place and time convenient to the interviewee. The interview schedule underwent review regarding its’ intelligibility for participants from non-first language English backgrounds. The schedule was piloted amongst students from a non-study year group. Interviews were audio recorded and the interviewer noted observations both during and afterwards in a field note journal.

### Analysis

The interviewer manually transcribed all interviews within 48 h of their recording in an orthographic method, retaining all contextual and non-verbal interactions within the transcript [52] to ensure credibility. A local translator ensured accuracy during translation of occasional Nepali words employed by participants. Each interviewee received a summary of their interview transcript, in order to comment upon its’ accuracy. A single researcher undertook manual coding, using semantic codes, and thematic analysis (TA).

## Results

Thematic analysis of the interviews generated 40 codes, with 5 subthemes and 2 overarching themes relating to role modelling.

The majority of participants, when asked about effective or ineffective CS teaching, described incidents of observing clinicians displaying behaviors and skills either taught explicitly during C.S course or contrary to it. These incidents were coded as role-modelling.

- Theme 1: Positive Role modelling- with subthemes; Personal qualities, Inspiring
- Theme 2: Negative Role modelling - with subthemes; Good doctors but.., Contradictory Messages, How not to behave, Unprofessional behaviour, Affect-Emotional distress

### Theme 1; positive role- modelling

The majority of participants described role modelling incidents, many describing both positive and negative examples.

#### Personal qualities

Students particularly described observing doctors’ interactions with patients, highlighting their respectfulness. One student described witnessing a senior speaking kindly to a patient:

*“I have not seen doctors talking so kindly to patients like he…. he just sat in the chair and holding his hands and asked in Nepali “how are you?” “did you sleep well?” everything, “how is your appetite?” he was asking so kindly as if he was the son of that patient”* (Sonam, 4^th^ year).

This reflects other studies describing students’ attention to doctors’ respectfulness in clinical interactions [30]. Positive role-modelling includes; personal qualities, professionalism, teaching and clinical skills [36, 53, 54]. Personal qualities comprise many of those incorporated within PAHS’ CS curriculum; respect, compassion, honesty, empathy and patient-centred care. These are explicitly mentioned within PAHS’ core values and are employed as criteria for CS feedback and assessments. Many student participants identified these characteristics within their role modelling incidents. Highlighting particularly clinicians; rapport building, reassurance, kindness and respectfulness. One student reports:

**“***Dr* [name] *is one, the way he treat the patient is very very nice because he makes sure that he is building a rapport with the patient, that they understand what he is saying and he gives appropriate time to the patient, he is very patient.. every time he speaks the patient smiles because he makes the environment so friendly and so comfortable and they trust him and so the treatment also goes smoothly”* (Ram, 4^th^ year).

Ram continues, saying that he wishes to learn these skills from his role model:

*“it’s very good to see the doctor-patient relationship so good and so comfortable … I feel that I should learn that from him”* (Ram, 4^th^ year).

Some students identified specific learning from positive role modelling incidents. Several identified the need to give patients sufficient time to talk and the importance of listening:

*“I just think that we are always in rush in* [clinic]. *we have so many patients and we need to give some time, to just let them catch their breath first and understand what they have been going through”* (Khim,4th year).

Many students identified the positive impact their role models’ behaviors had on patients’ trust. Several described incidents in which patients initially refused an intervention, but subsequently consented after the clinician gained their trust:

“*Dr Y communicate with that patient and patient party* [relatives] *with very normal words. sir communicate so nicely… he said that “I will put it very slowly that it will not hurt you” and they trusted him and it was done successfully*” (Asim, 4^th^ year).

Several interviewees commented explicitly on learning CS from their role models.

“*So there are a few doctors that I have observed who have good communication with patient and how they treat patient, that is a thing that we should learn from … who treat the patient very nicely”* (Ram, 4^th^ year).

Several students described learning the value of ‘proper’ informed consent, with information given to the patient to enable decision making. Most interviewees identified their learning citing particularly; rapport building, polite greeting, reassurance, building and maintaining trust, listening and understanding patient’s perspectives. This reflects the strong influence of role models on learning [35] and development of patient-centered values [36], significant for health outcomes: compliance with treatment, pain control, symptom resolution, psychological and functional wellbeing [3–7]. Strong positive role-models are recognised as the vital ingredient in molding students’ professional identity as doctors [44] and impact their psychological well-being [48].

#### Inspiring

Several participants expressed their desire to be like their role models:

“*I thought maybe if I also had that skill I would be just as good as he is, the way patients from twenty-five years ago come and still want to get treated by him, just because he speaks so nicely, he makes the patient so comfortable*” (Khim, 4th year).

Another student described aspiring to be like their role model:

*“so I felt very good* [about the incident observed] *and I thought that if I could be like sir in some places and then it would be very good”,* (Sonam, 4^th^ year).

Our study participants identified these incidents as CS learning experiences. Not all role-modelling, however, was positive.

### Theme 2: negative role modelling

Many participants experienced very different role modelling from that discussed. All who cited positive role modelling also described a negative incident. The majority of participants related only negative incidents.

#### Good doctors but

Participants described witnessing senior doctors displaying poor communication skills:

*“so there are many good incident that we where we are very inspired of because of the CS of the seniors but there are also incidents where we just didn’t like the way they communicated with patient, the way they treated patient so there are both perspective*” (Ram, 4^th^ year).

Many students explained that they believed their seniors were good doctors but were not good at communicating:

*“even my senior doctors they are very good clinicians but they are not good communicators”* (Binod, Intern).

This participant implied that good communication was not necessary to be a good doctor. Our students’ experiences of negative role modelling reflect similar findings in other studies [55, 56]. Negative role models have a strongly detrimental impact on students [36]. Learners feel disappointed [57] and disillusioned at the belief that the patient-centred values taught, are idealistic and impossible in practice [25]. Learners may become demotivated and unconsciously develop cynical, arrogant attitudes and “disenchantment with medical practice” [58]. This adversely impacts their development of patient-centred values and professional behaviour [25, 57]. The majority of our study participants described such negative modelling incidents.

#### Conflicting messages

Many interviewees recounted observing behavior contradictory to explicit CS teaching.

*“not everyone learns about communication skills, how to talk with the patients and I have seen the doctor talk with the patient very rudely and they won’t even let them open up and talk about the problems”* (Shova, 2nd year).

Students reported feeling confused and misled by the conflicting messages received. Their expectations, raised during the course, were not reflected in the clinical reality they experienced, resulting in the confusion and disillusionment described in other studies [25, 59]. This has the potential to generate negativity towards both their role models and the medical profession in general, and to impact professional identity formation [38, 58]. Learners’ professional identity- their view of themselves as a doctor, is considered as vital as the knowledge and skills taught in medical school [60, 61].

Most of the negative incidents described were clinical, however, one student recounted a formal CS teaching session in which the behaviour modelled conflicted with explicit teaching:

“*we were advised not to read the presentation, not to read it line to line. but the teacher who was saying* [this] *was reading line to line. So it wasn’t quite effective in my opinion”* (Pritty, 2^nd^ year).

She then articulated the incongruity between the teaching and observation, questioning how she could learn:

*“we are taught a certain way but then we don’t see a certain behaviour, so it’s always contradictory in our mind. we are taught but we don’t see the people who teach us that behaving in certain ways like we are taught…. I don’t know, like how are we supposed to learn because retention is more of their way of teaching rather than we reading the slides”* (Pritty, 2^nd^ year).

#### Unprofessional behaviour

Several participants reported witnessing incidents of unprofessional behaviour. One student described observing a doctor being disrespectful towards an anxious patient, failing to reassure her:

*“I saw one of our teacher* [from CS course] *who is also a doctor, she was very rude to a patient. the patient was hesitant to open up her clothes and she was* [said] *“I’m the doctor” and she was very rude to her. she was not cooperating much because there was no rapport building probably”* (Shova, 2^nd^ year).

Another, recounted a doctors’ failure to divulge a cancer diagnosis to the patient or relatives prior to major surgery, causing distress and confusion:

*“due to the gastric carcinoma he had surgery…and I remember that her* [his] *daughter….was just outside the ICU room and she was complaining to the nurses that they didn’t know the disease at all.. already the surgery had undergone* [been performed] *… she was crying totally crying, her father was so ill and she was just waiting outside without knowing the disease” (Sonam, 4*^*th*^ *year).*

One student reported a doctor ignoring a patient’s wish not to move their injured arm, causing pain and distress:

*‘he just said that “I’m the doctor you are not. you have to do what I say” so he insisted*… *he just grabbed her arm and just gave her more pain so it wasn’t a good experience.. I just felt that it is very not a good way of treating patient who have come to us to get a treatment.”* (Ram, 4^th^ year).

These findings mirror other studies, with students observing unethical and unprofessional behaviour [62, 63] or “patient dehumanization” [35], with disrespectful or coercive treatment [62].

Students are continuously observing and learning, expecting clinical teachers to always behave professionally, noticing inconsistent or contradictory behaviour [30]. Observation of unprofessional and unethical behaviors, like those described, correlates with a higher chance of students behaving similarly [62]. A students’ belief that such behavior is acceptable increases with seniority [64].

One intern participant described observing a doctor rebuking a patient for delayed presentation and threatening to withhold treatment:

*“very immediate caesarean section was required and the doctors were angry, that was justified and they scolded to some extent, but they also went up to the extent of saying “we would no longer provide you services of caesarean section”* (Binod, intern).

Continuing, Binod attempted to justify the doctor’s poor behavior:

“*it was not totally the doctors’ fault but the doctor was frustrated*” (Binod, intern).

Some learners gradually develop respect for the negative, bullying and authoritarian culture and behaviors observed [35], possibly indicated by Binod’s comment. The explicit teaching of respect and empathy is undermined by the prevailing culture within the community of practice, which teaches “an ethic of detachment, self-interest and objectivity” [65], adversely affecting learners’ professional behaviour and identity formation [38, 48, 57].

#### How not to behave

All student participants articulated their desire to behave differently from their negative role models.

*“there were like instances where my seniors are dealing with angry parties, they also get angry and very irritated. so in those situations I don’t start saying you don’t have to do that, but I can realise what can be improved in that situations and what would I have done if I was in that situations”* (Bibek, Intern).

Wear and colleagues [66] reported that students are often disappointed by derogatory behaviour and conscious of not replicating it. In this way negative modelling can teach “how not to behave” and its’ negative consequences [30].

“*I felt very bad and actually I really think that I will not do this to any of my patients in the future*” (Shova, 2^nd^ year).

Many interviewees identified positive learning from negative role modelling incidents. One described learning the importance of information disclosure in reducing patients’ anxiety:

*“it taught me that communication is very very important. so that the patient know*[s] *about that disease, the prognosis of the disease, the surgery the side effects. it is very very important so that the patient’s anxiety, the fear regarding the disease or that prognosis it gets decreased****”*** (Sonam, 4^th^ year).

Another described observing a patients’ distress, caused through repeated attempts at a procedure. The student reflected on learning the importance of patients’ comfort and continued consent, of providing patient-centred care. One student articulated having learnt the value of patient privacy, particularly for stigmatising illnesses. Another, the significance of empathy and listening for building trust. In this way many were able to identify positive learning from negative incidents. Despite this, doubt remains as to the likelihood of this resulting in real life learning and behaviour change as the negative impact of such role modelling is known [35, 38].

#### Affect -emotional distress

Many participants reported emotional responses stimulated by negative role modelling incidents, coded under the subtheme of ‘affect’. Emotional responses differed between participants and included feeling; uncomfortable, unhappy, angry, helpless and empathising with patient’s feelings:

“*but if I was a patient party* [relatives] *or if I was a patient then I wouldn’t like that*” (Ram 4^th^ year).

One student empathised with patients’ sadness and disappointment, wondering if this would reflect badly on other doctors:

*“she must have felt very sad and very bad. she would probably would think that our hospital and the doctors here are probably rude like her and won’t want other relatives to feel the way she felt. she will probably say “please don’t go to Patan hospital because I was treated this way there”* (Shova, 2^nd^ year).

These comments mirror other findings of learners’ anger and overt resistance to the values observed [35].

Several students described feeling powerless to act when facing negative behaviour:

*“I felt very bad and I really wanted to say this…but we are students so we couldn’t say anything to that doctor*” (Shova 2^nd^ year).

These feelings of powerlessness reflect those of other studies [67] in which students felt disempowered and frustrated within their roles.

Another student expands on feeling helpless, articulating how this resulted in an internal conflict:

*“I couldn’t do anything because I thought that I am not the one to said* [say] *I should have said, maybe I should have said but I thought I am not the one…”* (Sonam, 4^th^ year).

This internal struggle and conflict is described in other studies [25]. Learners reported feeling angry and frustrated at their inability to prevent or stop unkind, unethical behaviour. There is a danger that students learn helplessness from such situations, particularly when no one within the healthcare community, intervenes to prevent or mitigate such behaviours.

Negative role modelling is observed by our students in Nepal, reflecting the significance of this issue for medical education around the world [26, 27, 31].

In reality life is more mixed. Role models cannot usually be characterised as either positive or negative, instead most demonstrate a combination of both in any encounter [27].

## Discussion on role models

Role-models teach and inspire by example whilst engaged in something else [26]. Role-modelling occurs in various contexts, anywhere a clinical teacher is observed [27] and is mostly informal and unplanned [29]. It is clear that students pay close attention to doctors’ interactions with others, particularly noting their respectfulness and communication [30]. Our participants identified these incidents as CS learning experiences, even if the role models were unaware.

A 2013 systematic review [36] emphasised role-modellings’ value for medical learning. Passi et al. identified three studies that confirm modelling of personal characteristics and professionalism helps mold the growth, CS and professional character development of future doctors [68–70]. Role-models have a strong influence on students. Eager to learn, they watch clinicians’ actions, attitudes, and interactions with others [30] as peripheral participants within the community of practice [37], learning what it means to be a doctor [25], shaping their professional identity [38]. This influence should not be underestimated [71]. These experiences, observations and interactions, not formal teaching, are the most formative in influencing learning and development [72, 73]. Through a powerful process, students learn and adopt the beliefs and behaviours, values and ideals of their role-model [31–34]. Adapting consciously and unconsciously to mirror them [74], seeking membership and acceptance within the community of practice participants [37]. Role models have a strong impact on students’ formation of their professional identity [42, 43]. Our study shows the strong influence role-models have on CS [35] and patient-centered values [36] development. Role-modelling is a powerful tool in experiential learning, with positive modelling shaping future doctors. However, the impact of negative role-models must be considered [36].

Negative role-modelling is a widespread problem encountered by many students around the world. Less than 50% of clinical teachers are described as positive models [55] and half students deem them poor models of doctor-patient relationship [56]. Literature reports that students observe unethical and unprofessional behavior [62, 63] or “patient dehumanization” [35] similar to that described by our participants. Poor doctor communication is widespread [75] with roots in the negative attitudes and values of clinicians, permeating medical practice and culture [25]. This situation is well described in the west [25] and our study indicates a similar picture in Nepal.

Negative role-models have a strongly adverse impact on students [36]. Students expect clinical teachers to behave professionally, noticing inconsistent or contradictory behaviour [30] which conveys contradictory messages [25, 59]. This inconsistency create tension and internal conflict in students forming ideas of what it means to be a good doctor [25], modelling their identity as future doctors upon those within their practice community [38, 67]. Our study participants clearly articulated this tension and internal conflict, struggling with conflicting messages. Negative role modelling has significant clinical implications, adversely impacting trainees’ professional behaviour [57]. Students feel that patient-centred skills, learned earlier in training, are idealistic and impossible in practice, particularly when seniors model conflicting behaviour [25]. Other learners, however, are disappointed and conscious of not replicating the negative behaviour [66], as seen in our study. For some students it teaches “how not to behave” [30], although doubt exists as to the extent this determination to be different translates into a real difference in learning and behaviour. What has been demonstrated is the influence such experiences have by teaching instead that taught behaviour and attitudes can “legitimately be side-stepped” once practicing in the real world [76].

Our study demonstrates that negative role-modelling is experienced by students in Nepal, reflecting the big issue it is for medical education around the world [26, 27, 31].

It is important to remember, however, that students are not passive but “active agents of their own learning” [35]. Socialising factors of this implicit curriculum exert influence on future doctors, however, students contribute by deciding whether to accept and adopt, or reject the messages communicated [35]. Our interviews depict students trying to make sense of their experiences. This occurs through both unconscious and conscious activities; observation, reflection and reinforcement [27]. Active reflection plays a key role in converting unconscious impressions into conscious thought, which can be deciphered into values and behaviours [27]. Facilitating students’ critical reflection of negative role modelling incidents, may mitigate their unconscious adoption of such negative behaviours and attitudes.

This study demonstrates the need for clinical educators and tutors need to recognise the powerful influence they have over learners. Medical educators should support the development of positive, conscious CS role modelling in clinical tutors and hence student learning in clinical settings. By enhancing and strengthening critical reflection upon experiential CS learning it may be possible to enable learners to consciously and critically evaluate and internalise their learning.

Data regarding the contextual factors of role modelling incidents was not collected or explored in, this is a limitation of the study and area for potential future research.

## Conclusion

Western society is pressurising the medical community and hence medical educators, to resolve the problem of poor doctor-patient communication [75]. The roots of this are the negative attitudes and values of clinicians, which permeate medical practice and culture [25]. In this environment, malleable and enthusiastic students struggle to interpret a doctor’s role, from the conflicting models presented. This is the situation described in the west however, our study indicates a very similar picture exists here in Nepal.

Although this study was conducted in Nepal, the findings are of interest to medical educators across South Asia with similar cultural contexts; where CS teaching is in its’ infancy and is running counter directional to the prevailing medical culture and practice [77–79].

In 2015, MCI initiated competency-based CS teaching within its’ ATCOM module [19], in medical schools across India [18]. This is welcome progress as many have identified CS as a pressing need in India [17]. However, the continued underemphasis and piece-meal approach to CS training has produced sub-standard CS and the predominance of paternalistic consultation styles, despite patient preferences to the contrary [77–79]. One important criticism of India’s ATCOM is its’ implementation by existing faculty [17]. This concern reflects the awareness that CS teaching, both explicit and role modelled (implicit), requires different skills and approaches to other clinical subjects [80]. It is possible that a good on paper, competency-based CS curriculum, differs significantly from the one implemented and role modelled [81], as faculty struggling to adapt to patient centered CS teaching [78].

Whilst the expansion of CS teaching in India is encouraging, we are concerned that students’ experiential learning from clinical role models, may conflict with the explicit CS curriculum. Curriculum change alone is not sufficient, medical educators need to ensure that faculty are sensitised and supported in developing new teaching skills, something not commenced in most Indian medical institutions [17, 82, 83].

Role modelling is an effective tool for CS learning; however, this study demonstrates that it is not always positive. Medical schools need to strengthen students CS learning in clinical contexts, by supporting the development of clinical tutors in conscious role modelling and through facilitating student’s critical reflection on their experiences. This study also flags up the centrality of culture in reviewing and seeking evidence to inform educational initiatives or developments. It is vital that medical educators listen to students’ accounts of what they are really learning [35]. Acknowledging that *“the most important, indeed the only, thing we have to offer our students is ourselves. Everything else they can read in a book*.” [84].

### Study limitations and biases

Study interviews were conducted in English, the second language of most participants. Although English is used throughout training, participants may have felt more comfortable speaking their first language for discussion. A single researcher conducted the data analysis, however, the results were discussed by the research team. The sample size for the study was small, however, sample sufficiency was indicated by data saturation.

## Data Availability

The datasets used and/or analysed during the study are available from the corresponding author on request.

## Abbreviations

CS: Communication skills
PAHS: Patan Academy of Health Sciences
